# Generalized estimation of the ventilatory distribution from the multiple-breath nitrogen washout

**DOI:** 10.1186/s12938-016-0213-y

**Published:** 2016-08-02

**Authors:** Gabriel Casulari Motta-Ribeiro, Frederico Caetano Jandre, Hermann Wrigge, Antonio Giannella-Neto

**Affiliations:** 1 Pulmonary Engineering Laboratory, Biomedical Engineering Programme, COPPE, Universidade Federal do Rio de Janeiro, Rio de Janeiro, Brazil; 2 Department of Anesthesiology and Intensive Care Medicine, University of Leipzig, Leipzig, Germany

**Keywords:** Pulmonary function tests, Ventilatory distributions, Multiple-breath washout, End-expiratory lung volume, Functional residual capacity, Dead space, Nitrogen, Ventilation to volume, Tikhonov regularization, Common dead space

## Abstract

**Background:**

This work presents a generalized technique to estimate pulmonary ventilation-to-volume (v/V) distributions using the multiple-breath nitrogen washout, in which both tidal volume (*V*_*T*_) and the end-expiratory lung volume (EELV) are allowed to vary during the maneuver. In addition, the volume of the series dead space (*v*_*d*_), unlike the classical model, is considered a common series unit connected to a set of parallel alveolar units.

**Methods:**

The numerical solution for simulated data, either error-free or with the N_2_ measurement contaminated with the addition of Gaussian random noise of 3 or 5 % standard deviation was tested under several conditions in a computational model constituted by 50 alveolar units with unimodal and bimodal distributions of v/V. Non-negative least squares regression with Tikhonov regularization was employed for parameter retrieval. The solution was obtained with either unconstrained or constrained (*V*_*T*_, EELV and *v*_*d*_) conditions. The Tikhonov gain was fixed or estimated and a weighting matrix (WM) was considered. The quality of estimation was evaluated by the sum of the squared errors (SSE) (between reference and recovered distributions) and by the deviations of the first three moments calculated for both distributions. Additionally, a shape classification method was tested to identify the solution as unimodal or bimodal, by counting the number of shape agreements after 1000 repetitions.

**Results:**

The accuracy of the results showed a high dependence on the noise amplitude. The best algorithm for SSE and moments included the constrained and the WM solvers, whereas shape agreement improved without WM, resulting in 97.2 % for unimodal and 90.0 % for bimodal distributions in the highest noise condition.

**Conclusions:**

In conclusion this generalized method was able to identify v/V distributions from a lung model with a common series dead space even with variable *V*_*T*_. Although limitations remain in presence of experimental noise, appropriate combination of processing steps were also found to reduce estimation errors.

**Electronic supplementary material:**

The online version of this article (doi:10.1186/s12938-016-0213-y) contains supplementary material, which is available to authorized users.

## Background

The multiple-breath nitrogen washout (MBN_2_W) and the single-breath washout are classical pulmonary function tests, based on the measurement of the concentration or fraction of N_2_ in the breathing gases, to evaluate the ventilation inhomogeneity. Among measures and indices obtainable from the MBN_2_W, one of the most useful is the functional residual capacity (FRC) or end-expiratory lung volume (EELV), meaning the relaxed lung functional volume comprising all ventilated alveolar units plus the series dead space. The MBN_2_W is also able to convey information about ventilation-to-volume (v/V) inhomogeneities, by profiling the rate of expiratory elimination of the tracer gas along many respiratory cycles [1]. Several indices developed to evaluate the ventilation inhomogeneity are derived from the MBN_2_W, such as the lung clearance index [2] and the multiple-breath alveolar mixing inefficiency [3]. However, different patterns of inhomogeneity may be represented by the same values of these indices, which limit their specificity. Despite being relatively benign and non-invasive, the MBN_2_W needed, when it was being experimentally explored, costly, bulky machinery such as respiratory mass spectrometers (RMS); it could not map anatomy nor the spatial distribution of the quantities it measured, and ultimately it saw a decline of interest as a technique for general, day-to-day clinical application.

Along the time, the instrumentation to measure respiratory gas concentrations underwent evolutions. From the huge RMS to tiny capnometers and oximeters, their price and size shrunk, warranting broader use for them as alternatives to a direct measurement of N_2_. The presence of such accessible devices suggest revisiting techniques such as MBN_2_W. Even the usually nontrivial correction of the asynchrony between flow and concentration signals inherent to sidestream gas sampling, which requires high accuracy and is mandatory for adequate measurements of breath-by-breath volumes of gases [4] can be spared, for instance with the available mainstream gas sensors [5] or by simplifications based in taking end-tidal values as representative of mean alveolar concentrations, hence obviating the need for synchronization algorithms [6, 7].

On the other hand, the mathematical models available for the analysis of v/V distribution from the MBN_2_W required very stringent maneuvers and circumstances: the fraction of N_2_ should decrease in an abrupt step between two respiratory cycles; tidal volume (*V*_*T*_) and, more importantly, the EELV should be kept constant throughout the maneuver, that is, inspiratory and expiratory *V*_*T*_ must be equal. Those requirements may be difficult to meet, especially when the subject breathing spontaneously or in assisted modes of mechanical ventilation (MV) cannot cooperate by keeping a steady respiratory pattern. In order to overcome these limitations, new mathematical models, and model fitting techniques, must be developed. The objective of this work is to present, with simulated signals, a novel, more general mathematical model for the MBN_2_W, which accounts for the presence of a series dead space [8] and possibly varying *V*_*T*_, EELV and inspiratory fraction of N_2_, accompanied by candidate data-fitting algorithms aiming at computing the v/V distribution in the lungs.

## Methods

**Fig. 1:**
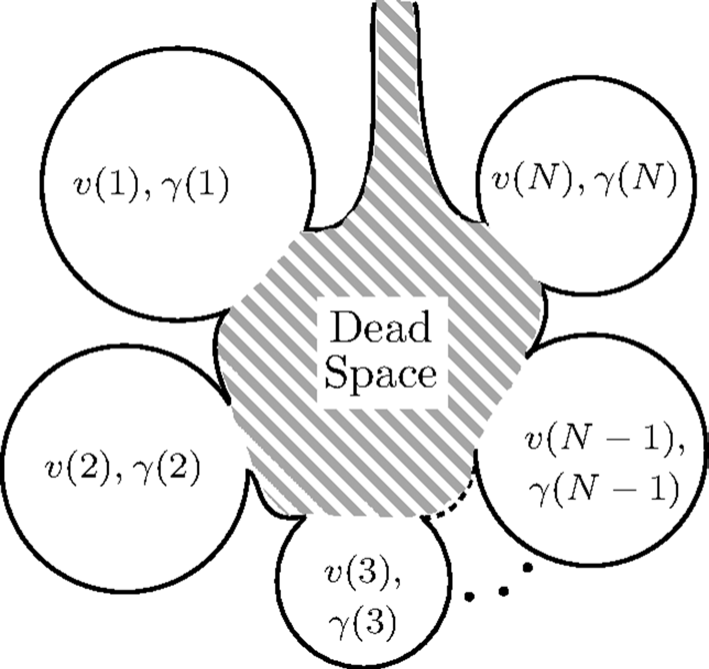
Model of the lung. The lung is composed of a common series dead space (hatched area) and a set of *N* alveolar parallel units. Each alveolar unit has its end-expiratory volume (*v*) and is ventilated by a fraction of the tidal volume ($\gamma$). Units communicate only through the dead space

The present mathematical model (Fig. 1) comprises a set of *N* parallel alveolar units with a common series dead space operating as ideal gas mixers. In this model, the sum of the fractions of *V*_*T*_ ventilating the alveolar units is equal to one, which differs from other approaches [1, 9] that model the anatomical dead space as one of the parallel alveolar units.

The assumptions governing the equations of the present model are the following:

1. Each alveolar unit, as well as the dead space, are ideal gas mixers;
2. All alveolar units are in parallel and they communicate exclusively over the common dead space;
3. Respiratory gases enter and leave all alveolar units synchronously and their volumes are never emptied;
4. All units, including the dead space, are in equilibrium and have the same N_2_ concentration prior to the nitrogen washout or washin;
5. The common dead space, whose volume is always known, is in series with the set of alveolar units, and includes the anatomical and the instrumental dead spaces;
6. The EELV, representing the sum of the volumes of all ventilated alveolar units plus the common dead space, is also always known;
7. Gas diffusional phenomena is negligible;
8. The respiratory exchange ratio is always unitary;
9. The N_2_ solubility in blood and tissues is negligible;
10. The ventilation-to-volume ratios of the alveolar units are logarithmically distributed.

These assumptions, except that concerning the presence of a series common dead space, although not necessarily as explicit in this form, are the same as in previous works [9, 10].

### The model formulation

Considering that a lung alveolar unit ($J$) is an ideal gas mixer characterized by an end-expiratory volume $v(J)$ and a ventilation fraction of *V*_*T*_$\gamma (J)$ the specific ventilation is defined as

$$S\left( J \right) = \frac{{\gamma \left( J \right)V_{T} }}{v\left( J \right)}.$$

By assuming an ideal step to a null inspiratory concentration of the tracer gas at the onset of the MBN_2_W and a constant EELV during the maneuver, the classical modelling approach calculates the alveolar unit concentration of N_2_ at each new breath $(k)$ as

1 $$F_{{N_{2} }}^{A} \left( {J,k} \right) = \frac{{F_{{N_{2} }}^{A} \left( {J,k - 1} \right)}}{1 + S\left( J \right)} ,$$

a dilution of the previous concentration in the end-inspiratory alveolar unit volume.

If the lung is composed by *N* parallel alveolar units, the end-tidal N_2_ concentration measured at the mouth is then a mixture

2 $$F_{{N_{2} }}^{et} (k) = \mathop \sum \limits_{J = 1}^{N} \gamma (J) F_{{N_{2} }}^{A} (J,k)$$

of the content of each alveolar unit weighted by the corresponding ventilation fraction. For a given distribution of $S(J)$ and a set of initial alveolar units concentrations the model in Eqs. 1 and 2 can be fitted to real washout data using a linear solver to estimate the corresponding $\gamma \left( J \right)$ values.

During MV, the ideal step condition is difficult to achieve without a specially designed circuit and does not hold with usual MV circuits. However, this condition can be disregarded by including the alveolar units inspired N_2_ concentration ($F_{{N_{2} }}^{I,A} \left( k \right)$) to the model. The new equation becomes

3 $$F_{{N_{2} }}^{A} \left( {J,k} \right) = \frac{{F_{{N_{2} }}^{I,A} \left( k \right)S\left( J \right) + F_{{N_{2} }}^{A} \left( {J,k - 1} \right)}}{1 + S\left( J \right)} ,$$

which has the same properties of Eq. 1. There is no explicit condition over $F_{{N_{2} }}^{I,A} \left( k \right)$, so this can be used to incorporate a common series dead space ($v_{d}$) to the alveolar units.

At the beginning of inspiration, the concentration of N_2_ in $v_{d}$ is equal to the end-tidal concentration of the previous breath (Eq. 2). The alveolar units inspired gas is a mixture of the ventilator delivered gas ($F_{{N_{2} }}^{I}$) and $v_{d}$ content, being given by

4 $$\begin{aligned} F_{{N_{2} }}^{I,A} &= \frac{{F_{{N_{2} }}^{et} \left( {k - 1} \right)v_{d} + F_{{N_{2} }}^{I} \left( k \right)\left( {V_{T} - v_{d} } \right)}}{{V_{T} }} \\ & = \left( {F_{{N_{2} }}^{\text{et}} \left( {k - 1} \right) - F_{{N_{2} }}^{I} \left( k \right)} \right) \cdot \frac{{v_{d} }}{{V_{T} }} + F_{{N_{2} }}^{I} \left( k \right).\end{aligned}$$

During MV, $V_{T}$ is usually constant but novel modes, markedly the variable ventilation, may provide different volumes for each breath. To extend the model while preserving the constant $S(J)$ distribution, a constant reference value of $V_{T}$ ($V_{T0}$) must be incorporated. Redefining the specific ventilation as

$$S\left( J \right) = \frac{{\gamma \left( J \right) \cdot V_{T0} }}{v\left( J \right)} ,$$

changes Eq. 3 to

5 $$F_{{N_{2} }}^{A} \left( {J,k} \right) = \frac{{F_{{N_{2} }}^{I,A} \left( k \right) \cdot \frac{S\left( J \right)}{{V_{T0} }}V_{T} \left( {\text{k}} \right) + F_{{N_{2} }}^{A} \left( {J,k - 1} \right)}}{{1 + \frac{S\left( J \right)}{{V_{T0} }}V_{T} \left( k \right)}} ,$$

which is directly dependent on the ratio $V_{T} /V_{T0}$. This ratio indicates that different *V*_*T*_ can decrease or increase the dilution rate compared to the reference. In practice, this shifts the γ(J) distribution to slower or faster compartment regions while preserving its shape. The reference *V*_*T0*_ can be freely chosen and is a way to normalize the distribution to better compare intra and inter subject data. The algorithm was designed to estimate $\gamma \left( J \right)$ irrespective of *V*_*T*_, though, so that we would have the same estimate for $\gamma \left( J \right)$ whether *V*_*T*_ is constant or variable. More generally, the intended effect of the algorithm is to factor out any change in *V*_*T*_ in order to have a single curve for $\gamma \left( J \right)$, for whatever values of *V*_*T*_, given a *V*_*T0*_.

In all previous equations the inspiratory ($V_{T}^{I}$) and the expiratory ($V_{T}^{E}$) tidal volumes were considered equal, as expected for a relaxed patient under MV. However, in case of assisted ventilatory modes or during spontaneous ventilation this assumption is no longer valid. As $S(J)$ is related to the (variable) alveolar units volumes, it needs to be defined using a reference value and the washout model must track the changes in EELV and $v(J)$.

The EELV changes are tracked by the ratio of the difference between $V_{T}^{I}$ and $V_{T}^{E}$ and the current EELV,

6 $$\beta \left( k \right) = 1 + \frac{{\left( {V_{T}^{I} (k) - V_{T}^{E} (k)} \right)}}{EELV(k)}.$$

To model the effect of EELV changes in each alveolar unit, in order to keep a unique shape for the distribution of ventilation and prevent alveolar units closures, we hypothesized that all alveolar units reduce or increase volume by the same ratio. Assuming that the reference for $v(J)$ is the volume at the beginning of the washout ($v_{0} (J)$), at any breath cycle a alveolar unit’s volume is known from the relation

7 $$v\left( {J,k} \right) = \beta \left( {k - 1} \right)v\left( {J,k - 1} \right) = \mathop \prod \limits_{i = 1}^{k - 1} \beta \left( i \right) v_{0} (J) .$$

The alveolar unit $N_{2}$ concentration is then calculated defining $S\left( J \right)$ in terms of $v_{0} \left( J \right)$. This leads to

8 $$F_{{N_{2} }}^{A} \left( {J,k} \right) = \frac{{F_{{N_{2} }}^{I,A} \left( k \right) \cdot \frac{S\left( J \right)}{{V_{T0} }}V_{T}^{\text{I}} \left( {\text{k}} \right) + F_{{N_{2} }}^{A} \left( {J,k - 1} \right)\mathop \prod \nolimits_{i = 1}^{k - 1} \beta (i)}}{{\mathop \prod \nolimits_{i = 1}^{k - 1} \beta (i) + \frac{S\left( J \right)}{{V_{T0} }}V_{T}^{I} \left( k \right)}} ,$$

which reduces to Eq. 5, if $V_{T}^{E} = V_{T}^{I}$ for all breath cycles, and to Eq. 3, if in addition the $V_{T}$ is fixed. Considering the assumption that $\beta \left( k \right)$ is equal for all alveolar units, the end-tidal expired gas must account for the volume kept or released every breath cycle. Therefore, the end-tidal gas is now

9 $$F_{{N_{2} }}^{et} \left( k \right) = \mathop \sum \limits_{J = 1}^{N} \gamma \left( J \right)\frac{{\left( {V_{T}^{I} \left( {\text{k}} \right) + \left( {1 - \beta \left( k \right)} \right)\mathop \prod \nolimits_{i = 1}^{k - 1} \beta (i)\frac{{V_{T0} }}{S\left( J \right)}} \right)F_{{N_{2} }}^{A} \left( {J,k} \right)}}{{V_{T}^{E} \left( k \right)}} ,$$

With $\gamma \left( J \right)V_{T0} /S\left( J \right)$ expressing $v_{0} \left( J \right)$. Equation 9 is simplified to Eq. 2 for all the simple cases and then Eqs. 8 and 9 are a unified model for lung alveolar unit washout. It is important to note that in the most general case, with variable $V_{T}^{I}$ and $V_{T}^{E}$, the estimated $\gamma \left( J \right)$ distribution is the one at the beginning of the washout maneuver, if $V_{T} = V_{T0}$ is considered.

For the present numerical results, the MBN_2_W was simulated for a human adult under MV. The EELV ranged from 1.0 to 4.244 L [11, 12] with N = 50 alveolar units log-distributed on $S\left( J \right)$ in the range of 0.01–100. Mean *V*_*T*_ was 0.5 L [13] and was kept constant or with a variability of 25 % [14]. The $v_{d}$ was of 0.125 L [15].

### Recovering the distributions: inverse problem formulation

To define the inverse problem of finding $\gamma \left( J \right)$ knowing $F_{{N_{2} }}^{et}$ at each breath cycle of washout, consider the vector form of Eq. 9

10 $$F_{{N_{2} }}^{et} \left( k \right) = \left[ {a_{1} \cdots a_{j} \cdots a_{N} } \right]\left[ {\begin{array}{*{20}c} {\gamma \left( 1 \right)} \\ \vdots \\ {\gamma \left( N \right)} \\ \end{array} } \right], \;\;\;a_{j} = \frac{{\left( {V_{T}^{I} \left( {\text{k}} \right) + \left( {1 - \beta \left( k \right)} \right)\mathop \prod \nolimits_{i = 1}^{k - 1} \beta (i)\frac{{V_{T0} }}{S\left( j \right)}} \right){\text{F}}_{{{\text{N}}_{2} }}^{A} \left( {j,\;k} \right)}}{{V_{T}^{E} \left( k \right)}}.$$

With Eq. 10, it is possible to see that the recovery of the vector $\gamma$ is the solution of a system of linear equations. It can be noted that even with zero noise the inverse problem is most likely ill-posed when the number of alveolar units is larger than the number of washout breath cycles. To get a unique, smooth solution, the regularization method of Tikhonov [16] can be applied and up to three constraints over $\gamma$ can be added.

The first constraint is the classical nonnegativity condition [1, 9]. The second is the constraint of unitary total ventilation [9]. Finally, the sum of alveolar units volumes is constrained to $EELV - v_{d}$.

The problem of finding $\gamma \left( J \right)$ becomes equivalent to solve a constrained nonnegative least-square problem

11 $$\mathop {\hbox{min} }\limits_{\gamma } ||A\gamma - b||^{2} + ||\lambda L\gamma ||^{2} , \;s.t.\gamma \ge 0 ; \;C\gamma = c ,$$

where $A \in {\mathbb{R}}^{MxN}$ is a matrix whose elements $a_{ij}$ are the predicted $N_{2}$ concentration of alveolar unit $j$ at breath cycle $i$ and $b \in {\mathbb{R}}^{M}$ is the measured $F_{{N_{2} }}^{et}$ during the washout maneuver. The second term imposes a smoothness condition with a scalar gain λ and a weighting matrix $L \in {\mathbb{R}}^{NxN}$, which can be used to penalize some alveolar units more than others. Matrix $C$ is the constraint matrix.

See the Additional file 1 for a summary of the algorithm to estimate the distribution of ventilation.

### Least square solvers

The complete least square problem with three constraints was solved using an active-set algorithm [17], while in the condition requiring only nonnegativity, the Lawson and Hanson algorithm was used [18]. In either cases, the Tikhonov smoothness was applied expanding the matrix $A$ and vector $b$ with $\lambda L$ and a null vector, respectively.

### Sensitivity to noise

**Fig. 2:**
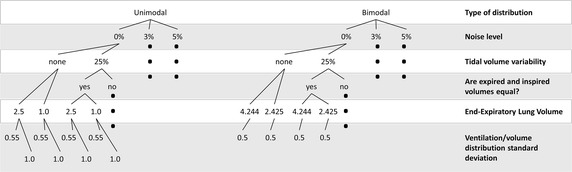
Scheme of simulations. Unimodal and bimodal distributions with error-free or noisy data and with tidal volume variability (or not). End-expiratory lung volume as well as distribution standard deviation were selected from two options. *Black dots* indicate that flow chart continuation coincides with the correspondent at the same level

Sensitivity to noise was assessed by the numerical simulation of uni- and bimodal log-normal distributions with different log-means and log-standard deviation (logSD) [1, 9]. Unimodal shapes are typical of normal young subjects and bimodal shapes are found in normal old as well as in patients with cystic fibrosis or other obstructive lung diseases [1]. The simulations included washouts with error-free $F_{{N_{2} }}^{et}$ and measurements corrupted by normal distributed noise with zero mean and 3 or 5 % standard deviation(SD) [9]. The three $V_{T}$ conditions ($V_T^E=V_T^I=constant$; $V_{T}^{E} = V_{T}^{I} \ne constant$; and $V_{T}^{E} \ne V_{T}^{I} )$ have been considered with a basal $V_{T}$ of 500 mL. In presence of noise or with non-constant $V_{T}$ the simulations were repeated 1000 times. At the beginning of the washout, instead of an ideal step change, the N_2_ concentration fell to zero at the fifth breath cycle, simulating the washout of the inspiratory external circuit of the mechanical ventilator. Figure 2 depicts a diagram of all simulated conditions.

### Reconstruction conditions

**Fig. 3:**
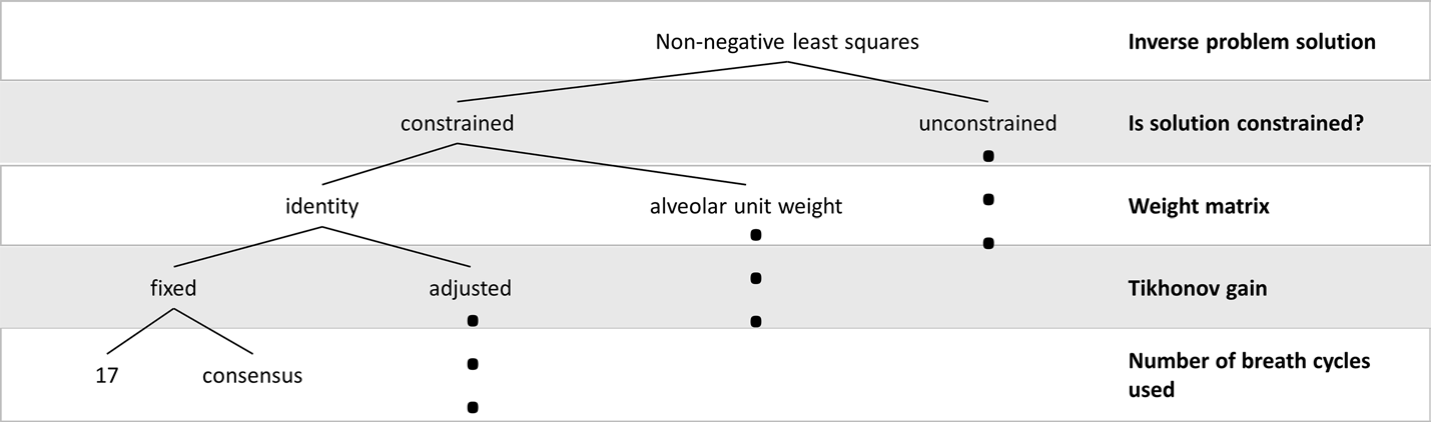
Distributions recovery flow chart. Nonnegative least squares are used either unconstrained or constrained (imposed values for tidal volume, end-expiratory lung volume and series dead space) considering a chosen weighting matrix, a Tikhonov gain and different number of breath cycles. *Black dots* indicate that flow chart continuation coincides with the correspondent at the same level

To assess the effect of noise on different reconstruction approaches, simulations covered the nonnegative and the full-constrained least square problems, 17 breath cycles [9] or consensus washout (N_2_ concentration at the end of the of the washout at 1/40 of the starting concentration [4]) and the use of $L$ equal to identity or the alveolar unit weighting matrix (WM) proposed by Lewis et al. [1]. Additionally, two Tikhonov gains $\lambda$ have been used. One gain was constant, independent of noise and equal to 0.033, the other was calculated for each simulated washout as the maximum between this constant value and three values estimated by heuristic methods (l-curve, generalized cross validation and normalized cumulative periodogram) [19]. Analogously, for noise-free simulations, the constant $\lambda$ was equal to 0.0008 and the estimate was the maximum among this value and the heuristics. Figure 3 shows a diagram of the recovering flow chart.

### Figures of merit

The quality of estimation was assessed by the SSE between the reconstructed and reference distributions and a set of parameters characterizing the distribution shape.

The shape parameters are the first three moments: mean, variance (expressed by the logSD) and skewness, the latter calculated only for unimodal distributions. All moments were estimated considering a continuous probability density function $P\left( x \right)$, with $x = log(S)$. The figures of merit are the differences between the parameters calculated for estimated and reference, expressed as a percentage of reference for mean and logSD. Results are calculated as mean and SD of the absolute values of all simulations. Additionally, we established an objective characterization of the reconstructed waveform shape. A unimodal shape was defined as the distribution that has only one peak or, in the presence of two peaks, the smaller to larger peak ratio must be less than 20 % or they must be spaced by less than five alveolar units. A bimodal shape was the distribution which has two peaks with smaller to larger peak ratio above 30 % separated by a valley of at most 80 % of smaller peak amplitude. This classification was applied to all tests performed.

For a visualization of the reconstructed curves, they are plotted as boxplot of all repetitions for the best combination of smoothness gain, weighting matrix and least square solver. All software routines were written and run in MatLab (MathWorks, USA).

## Results

**Fig. 4:**
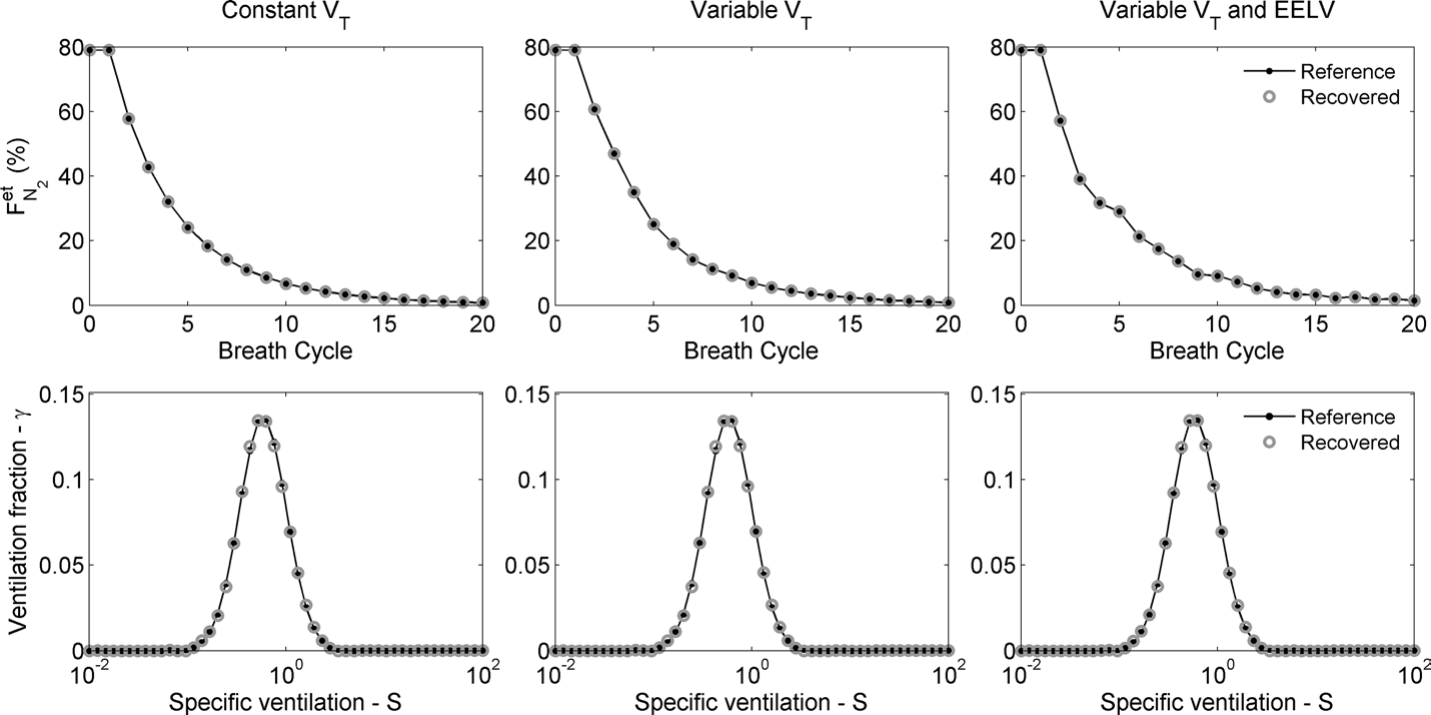
Examples of washout for the three tidal volume (V_T_) conditions. *Left panel* *V*_*T*_ is constant; *central panel* *V*_*T*_ is variable but end-expiratory lung volume (EELV) is constant; *right panel* variables *V*_*T*_ and *EELV*. *Closed circles* are reference and *open circles* are recovered values. $F_{{N_{2} }}^{et}$ is the *N*_*2*_ end-tidal fraction

Figure 4 shows examples of N_2_ washouts from ambient air to 100 % O_2_ for the three *V*_*T*_ conditions. The simulations were accomplished with error-free data. The v/V distributions were identical for the three conditions. Note that for the most general case (variable *V*_*T*_ and *EELV*), the $F_{{N_{2} }}^{et}$ can increase along the washout when $V_{T}^{E} > V_{T}^{I}$. Recovered $F_{{N_{2} }}^{et}$ and v/V distributions were practically coincidental with the simulated.

**Fig. 5:**
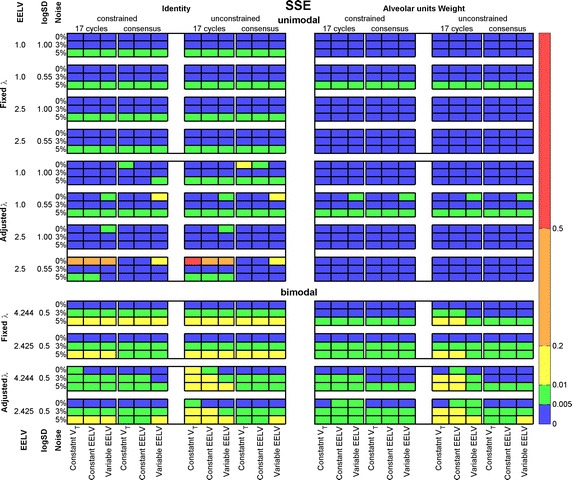
Sum of squared error (SSE) between estimated and reference ventilation-to-volume distributions. Unimodal (*upper panel*) and bimodal (*lower panel*) ventilation-to-volume distributions. All simulation and recovery conditions are depicted and indicated (rows and columns of the matrices). *Colors* coded on the *right side* of the figure indicate intervals of values of the mean SSE; *EELV* end-expiratory lung volume, *V*_*T*_ tidal volume

Figure 5 presents the SSE means for the recovered unimodal and bimodal distributions, considering either the first 17 breath cycles or the consensus washout maneuver (see the SD in Additional file 1: Figure S1). Small values of SSE (colored blue) represent estimated distributions close to the reference with alveolar units outside the simulated $S(J)$ span having a small ventilation fraction. The best result was achieved with fixed λ, and with alveolar unit weight (upper right panel). The constrained and unconstrained solutions gave similar results, slightly better with the former for bimodal distributions. The number of breath cycles used for recovering had minor effect on the results. Regarding the consensus option, the minimum number of breath cycles taken for v/V identification was 15 (unimodal, EELV = 1.0 L, logSD = 0.55) and the maximum was 50 (bimodal, EELV = 4.244 L, logSD = 0.50 for both modes).

**Fig. 6:**
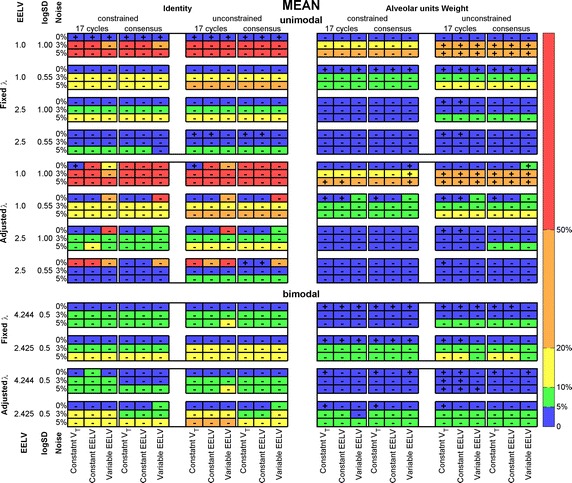
Averaged absolute values of the relative difference between means of estimated and reference ventilation-to-volume distributions. Unimodal (*upper panel*) and bimodal (*lower panel*) distributions. All simulation and recovery conditions are depicted and indicated (rows and columns of the matrices). *Colors* coded on the *right side* indicate intervals of the relative difference; sign inside each cell indicate if the recovered distribution is right (+) or left (−) shifted. *EELV* end-expiratory lung volume, *V*_*T*_ tidal volume

The averaged absolute values of the relative errors of the means of unimodal and bimodal estimated distributions are shown in Fig. 6 (see the SD in Additional file 1: Figure S2). A positive sign inside the cell indicates a tendency to estimate right shifted distributions, while a negative sign indicates a left shift. Analogously to the SSE evaluation, the best solution was found with fixed λ, with WM and constrained (up-right panel).

**Fig. 7:**
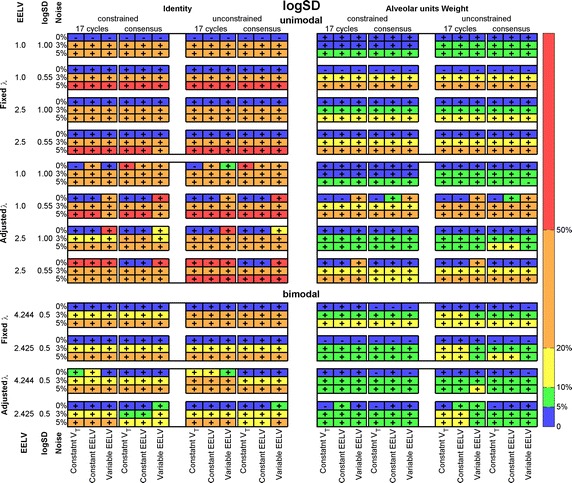
Averaged absolute values of the relative difference between logSD of estimated and reference ventilation-to-volume distributions. Unimodal (*upper panel*) and bimodal (*lower panel*). All simulation and recovery conditions are depicted and indicated (rows and columns of the matrices). *Colors* coded on the *right side* of the figure indicate intervals of the relative difference. Sign inside each cell indicate if the recovered distribution is broadened (+) or narrowed (−); *EELV* end-expiratory lung volume, *V*_*T*_ tidal volume

Besides displacement of the mean value, the distribution can be narrowed or broadened. This characteristic was measured with the relative errors of logSD, shown in Fig. 7 (see the SD in Additional file 1: Figure S3). Positive signs indicate broadened and negative signs, narrowed distributions. Signs, except for a few error-free data distributions, were positive (broadened). Analogously to the precedent evaluations, the best results were found with fixed λ, WM and constrained (up-right panel). The errors increased for small logSD distributions.

**Fig. 8:**
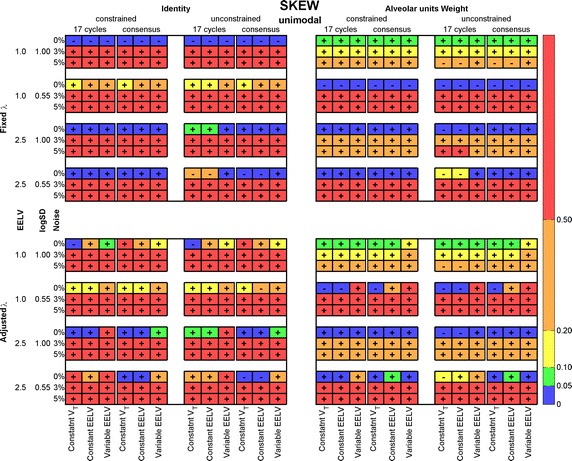
Averaged absolute values of the difference between skewness of estimated and reference unimodal ventilation-to-volume distributions. All simulation and recovery conditions are depicted and indicated (rows and columns of the matrices). *Colors* coded on the *right side* indicate intervals of the skewness error mean value; sign inside each cell indicate if the recovered distribution is tailed to the right (+) or left (−). *EELV* end-expiratory lung volume, *V*_*T*_ tidal volume

For a unimodal distribution, a shift in the mean can be a consequence of displacement of the entire distribution or a loss in symmetry. This was assessed with the difference of skewness in estimated and reference distribution, as shown in Fig. 8 (see the SD in Additional file 1: Figure S4). A positive (negative) sign inside the rectangle indicates a tail to the right (left). Note that the skewness sign was positive in most cases and opposed to the mean difference, which means an asymmetry of the estimated distribution. The errors were smaller than 0.05 only for error-free data, and increased tenfold when noise was added to the data.

**Fig. 9:**
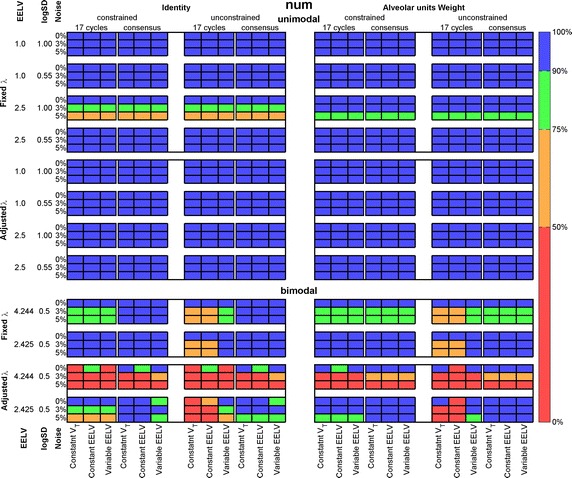
Waveform shape classification for unimodal (*upper*) and bimodal (*lower*) ventilation-to-volume distributions. All simulation and recovery conditions are depicted and indicated (rows and columns of the matrices). *Colors* coded on the *right side* indicate intervals of distribution shape agreements in percentage. *EELV* end-expiratory lung volume, *V*_*T*_ tidal volume

The waveform shape classification is shown in Fig. 9 for both unimodal and bimodal distributions. The results were calculated in percentage of agreements after 1000 tests for each case. For unimodal distributions, a high agreement was found for all recovering combinations. Considering 5 % of noise, the overall maximum agreement averaged 97.2 % (constrained, WM, adjusted lambda, 17 breath cycles) and the minimum was 84.0 % (constrained, identity, fixed lambda, consensus washout). For bimodal distributions the results spread from high to very low agreement. The maximum was 90.0 % (constrained, identity, fixed lambda, 17 breath cycles) and the minimum was 17.8 % (unconstrained, identity, adjusted lambda, 17 breath cycles).

**Fig. 10:**
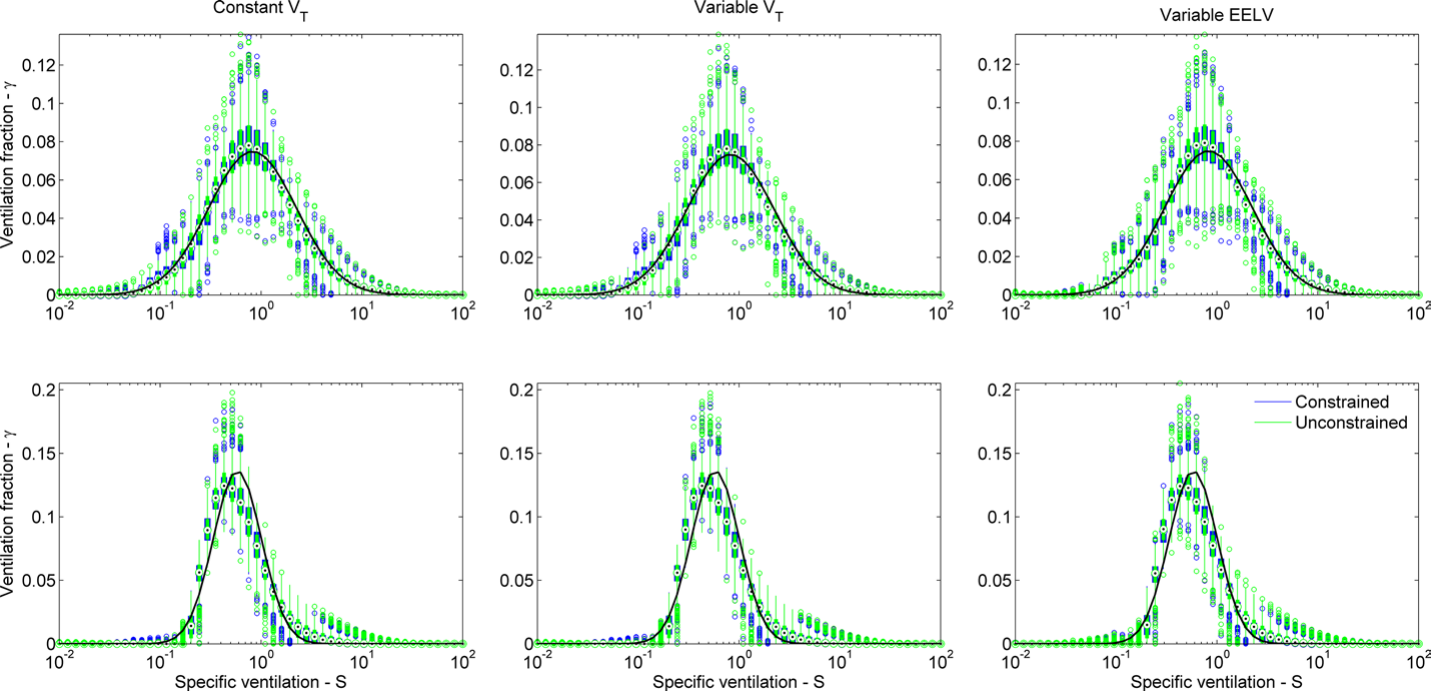
Boxplot of all estimated unimodal distributions with EELV = 1.0 and 5 % of noise. Only the alternative with alveolar unit weight, fixed Tikhonov gain and consensus washout is represented. Both constrained (*blue*) and unconstrained (*green*) linear solvers are shown. logSD = 1.00 (*upper panels*); logSD = 0.55 (*lower panels*). *Black dots* median values, *wide bars* first and third quartiles, *thin lines* extend to extreme values that are not outliers, *colored circles* outliers

**Fig. 11:**
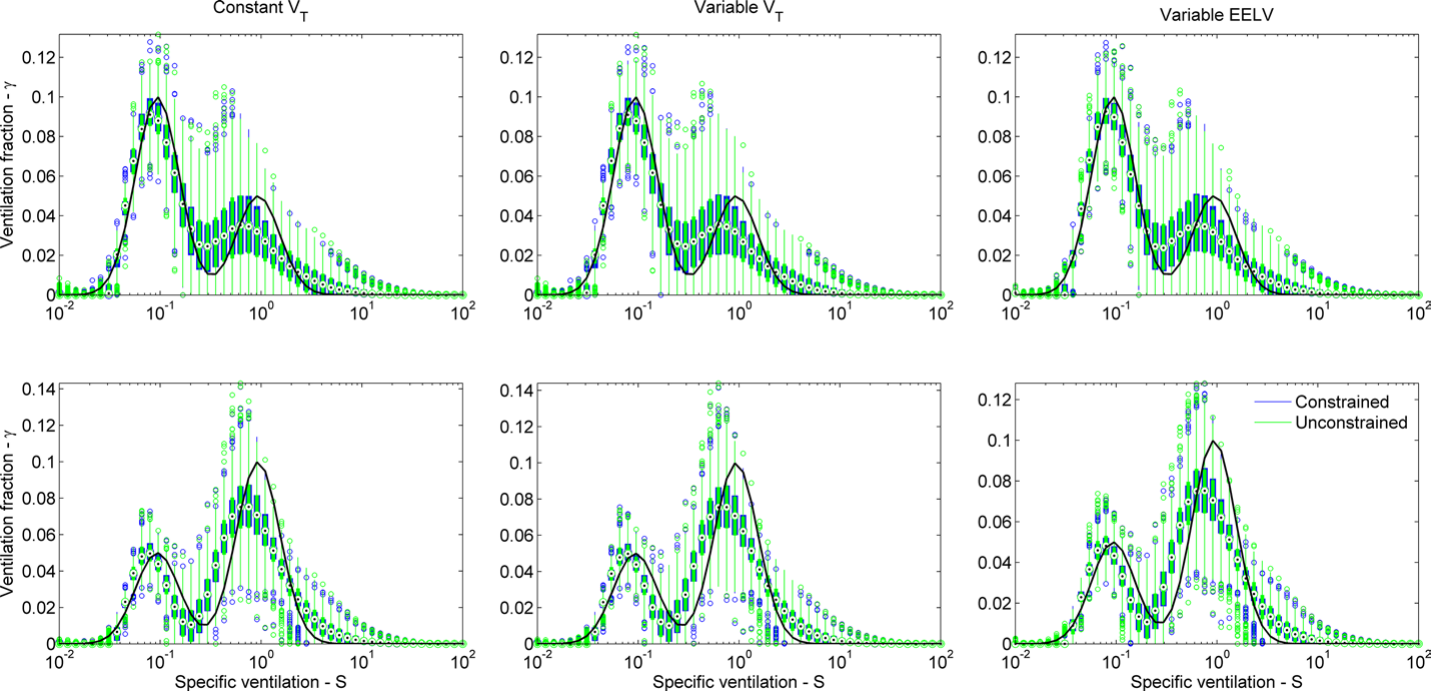
Boxplot of all estimated bimodal distributions with 5 % of noise. Only the alternative with alveolar unit weight, fixed Tikhonov gain and consensus washout is represented. Both constrained (*blue*) and unconstrained (*green*) linear solvers are shown. End-expiratory lung volume (EELV) of 4.244 L (*upper panels*) and 2.425 L (*lower panels*). *Black dots* median values, *wide bars* first and third quartiles; *thin lines* extend to extreme values that are not outliers, *colored circles* outliers

In order to depict the summary statistics and get a qualitative view of estimations, Figs. 10 and 11 show the boxplot of estimated unimodal and bimodal distributions, respectively. The plots represented the alternative of using WM, fixed Tikhonov gain, consensus washout estimation applied to data with 5 % of noise. In general, for unimodal distributions (Fig. 10) the median of the estimated alveolar unit fraction of ventilation was close to the reference, with higher agreement for the broad distributions (upper panels). For narrow distributions (lower panels), the estimated distribution has a small right skew, which leads to the negative difference of mean and positive skewness (Figs. 6, 8, respectively). There are also some outliers outside the simulated alveolar units span, which increase the estimated logSD (Fig. 7). Most of these outliers are in the fast region (high v/V), as an effect of noise, but do not reach the end of $S(J)$ range, penalized by the WM matrix. For bimodal distributions (Fig. 11), the estimated medians presented modes close to the reference with a higher agreement to the mode at low v/V. The high v/V mode was shifted to the left with a tail to the right.

**Fig. 12:**
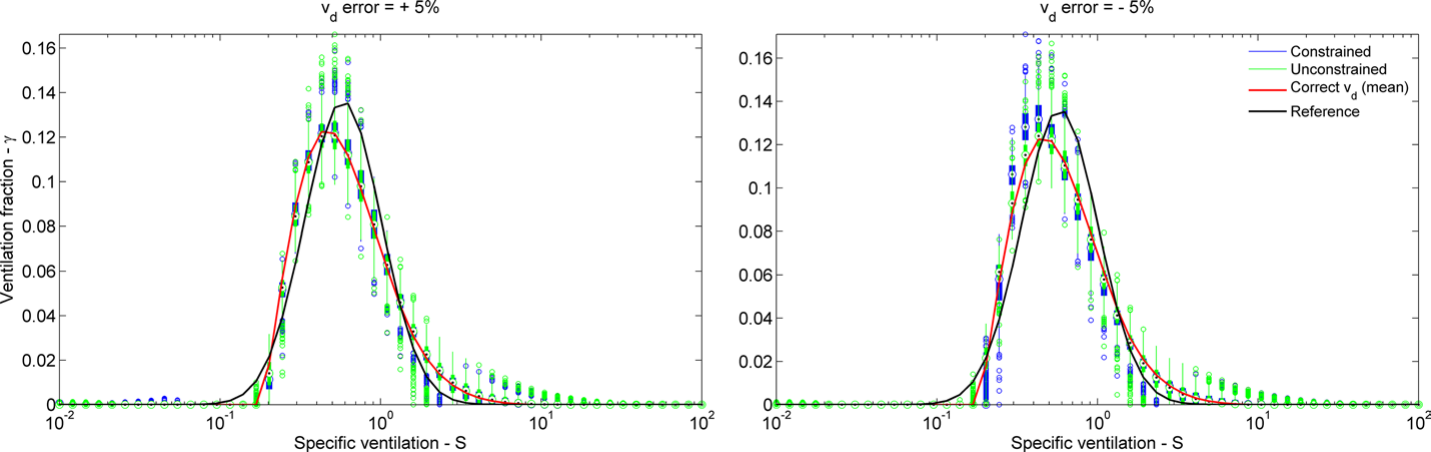
Influence of errors of dead space on the estimated distribution. Boxplot of all estimated unimodal distributions with EELV = 1.0 L, logSD = 0.55, 3 % of noise on N_2_ concentration and 5 % of error in dead space ($v_{d}$) volume estimation. Only the case with alveolar unit weight, fixed Tikhonov gain and consensus washout is represented. Both constrained (*blue*) and unconstrained (*green*) linear solvers are shown. Reference distribution (*black*) and estimated without $v_{d}$ error (*red*). *Black dots* median values, *wide bars* first and third quartiles, *thin lines* extend to extreme values that are not outliers, *colored circles* outliers

To assess the influence of errors of $v_{d}$ measurement on the estimated distribution, Fig. 12 shows the boxplot of a 3 % noisy unimodal distribution with EELV = 1.0 L and logSD = 0.55 estimated with +5 and −5 % of error on the simulated $v_{d}$. When compared with the estimated error-free $v_{d}$ (red line), an over/underestimated $v_{d}$ lead to a small right/left shift on the distribution, respectively.

## Discussion

A brief summary of the results ensues. A generalized equation has been demonstrated and tested which allows for the identification of v/V distributions for a lung with a common series dead space and variable *V*_*T*_ and EELV. As seen in Figs. 2 and 3, a rather large set of combinations of v/V distributions and strategies for the recovery algorithms was tested, mostly with results that support the proposed approach. Considering all *V*_*T*_ conditions and all figures of merit in ensemble (errors in SSE, mean, logSD, and skewness) the best combination for unimodal distributions was obtained with the joint use of WM, constrained solver, and fixed λ. The number of cycles used for recovery, 17 or consensus washout, produced similar outcomes. The best classification for unimodal distributions was achieved with WM, constrained solver, 17 cycles and adjusted (instead of fixed) λ and resulted in 97.2 % of agreement, higher than the best combination for moments’ estimates (91.6 %). For bimodal distributions, the best result for the figures of merit was obtained with WM, the constrained solver and adjusted (instead of fixed) λ, and the technique was insensitive to the number of breath cycles. The best classification was achieved with the identity matrix (instead of WM), the constrained solver and the fixed λ (90.0 %), much higher than using the best combination used for the moments’ estimates (63.9 %).

The use of Tikhonov regularization spans several fields of applied sciences, as an effective tool to enforce smoothness when a curve with such a characteristic is expected, as in the present case. Nevertheless, it requires choosing the shape of the corresponding matrix and its values; here, only two regularization matrices were tested, the identity matrix and the WM proposed by Lewis et al. [1] and applied as described by Whiteley et al. [9], which penalizes the non-zero solutions found close to the upper and lower limits of the specific ventilation. Albeit we have tested a numerous set of distributions and recovering combinations, this is far from exhaustive. Moreover, the v/V distributions were also chosen, in the present case, to represent both normal and abnormal lungs of adults [1], with 50 alveolar units as in almost all previous works on this subject. We used specific ventilation ranging from 0.01 up to 100, as did Wagner [20] and Kapitan [21], differently from Whiteley et al. [9]. Different choices may be required to model other patients e.g. infants.

As expected, all estimates were sensitive to measurement noise. The simulated experimental errors limit the recovery of v/V distributions with MBN_2_W, and that would be expected with real signals. It remains a challenge to increase the accuracy of measurements in order to identify, with a single test, whether the distribution is uni- or bimodal, skewed to right or left, narrow or broad and apply this to assess or to control therapeutic procedures. The classical experimental setup requires an RMS and the use of mean expired gas demands the synchronization of signals, which are additional sources of errors [22]. The current technology allows the indirect monitoring of $F_{{N_{2} }}^{et}$ as the complement of O_2_ and CO_2_ gas fractions measured by the sensors available in mechanical ventilators [23] or other instruments [7, 24], which may also involve errors. Moreover, the MBN_2_W, if performed with small amplitude changes of O_2_, for instance with critically-ill patients [25], may see a reduction in the signal-to-noise-ratio; however, since with the present model the N_2_ washout may be followed by a series of washin-washout maneuvers, perhaps the increased number of breath cycles may compensate for increased relative measurement errors.

The effect of experimental errors on recovering v/V distributions was studied by Lewis et al. [1], Kapitan [21] and Whiteley et al. [9] with noise levels of 1, 0.1 and 3–5 %, respectively. We consider the highest error limits more realistic, since other sources of errors such as that in flow rate and *V*_*T*_ must be considered. Their recovering algorithms were comparable to those applied in the present work [1, 9]. A direct comparison of results is difficult, however, since the modelled distributions, as well as the criteria to quantify the recovering estimates accuracy, are different. However, the present study used the same error amplitudes as tested by Whiteley et al. [9] and also evaluated the first moments and the agreement in curve shapes for both unimodal and bimodal distributions. In particular, our results of shape agreement were higher than reported by Whiteley et al. [9]. Nevertheless, these authors did not specify their criteria; our model allowed, for example, for the presence of a small second mode in order not to reject the identification of a unimodal distribution. Differently, our best classification result was found without the use of the WM of Lewis et al. [1].

The present model includes most of the assumptions proposed in previous works addressing the recovery of v/V distributions from the MBN_2_W [1, 9]. A key difference here is the use of a common series dead space, instead of a parallel dead space (all-parallel model). There is a correspondence between both models, already demonstrated by Evans [26]. Nevertheless, if the washout comes from a lung with a common series dead space and the v/V reconstruction is based on the all-parallel topology, the distributions will not be identical. One first obvious difference is the amplitudes of the fractional ventilations, which add up to one in our model, but to ($1{ - }{{v_{d} } / {V_{T} }}$) in the all-parallel model. This amplitude reduction will imply a shift to the left and a warped shape in the classical graphical representation of the v/V distribution. Actually, the all-parallel model is a representation of the fractional alveolar ventilation instead of the total ventilation (which includes dead space rebreathing). It is a matter of choice: the all-parallel model follows the gas exchange terminology, whereas our model quantifies the real volume change of each alveolar unit, comparable to image estimates of ventilation distribution [27]. The $v_{d}$ can be measured with the Fowler’s technique [28] and preferentially by capnography instead of N_2_ monitoring, because N_2_ fraction amplitudes fall quickly along the washout and the early N_2_ emptying of fast alveolar units tends to apparently increase the magnitude of $v_{d}$ [10].

It should be noted that some of the assumptions involved in the assembly of the present general equation are rather restrictive and some are opposed to evidence, for instance the synchronous filling and emptying of alveolar units, and homogeneous N_2_ concentrations in all units before the beginning of washout. Also, a simple model was used for $v_{d}$, a single airway connecting all alveolar units to the outside environment, disregarding the several ramifications of the airways and the inherent presence of personal dead space [29]. Some of the assumptions are needed because the information obtainable from the MBN_2_W is limited and simplification is mandatory.

The nonnegative least squares solution was obtained either with or without two additional constraints: (1) the *V*_*T*_ fractions add up to one; (2) the sum of the alveolar units volumes is equal to (${\text{EELV}}{ - }v_{d}$). Obviously, the effect on results of using these constraints depends on the accurate measurements of *V*_*T*_, EELV and $v_{d}$. Measuring EELV and *V*_*T*_ is inherent to the MBN_2_W. Regarding EELV, its accuracy depends on both the N_2_ analyzer and the flow rate sensor from which the *V*_*T*_ is calculated. Moreover, a time delay correction to synchronize both signals may be necessary and this is a critical step [4, 22]. During washout, an aliquot of N_2_ comes from blood and tissues and must be subtracted from the total eliminated amount of N_2_ for the correct EELV evaluation [30]. However, the absence of reliable data has induced the recommendation not to correct for this effect [4]. Additionally, it was shown that the N_2_ diffusion from outside the lungs causes only a small effect on v/V distributions recovering [31]. It must be noted that the present technique is not limited to the use of N_2_ as the test gas. Other inert gases such as SF_6_ or He [4] can be used and with obvious advantages because of their lower solubility in water and body tissues. Another potential confounder arises from the fact that, during washout, the composition of gases changes breath-by-breath and usual flowmeters are not immune to dependencies on physical properties of the gas mixture such as viscosity, density or thermal conductivity, depending on the operating principle of the transducer.

## Conclusions

In conclusion, we demonstrated a generalized equation that allows the identification of v/V distributions without *V*_*T*_ and EELV restrictions, for a lung model with a common series dead space. Moreover, nonidealities such as the departure from a step change in N_2_ concentration are allowed by the model, which supports the applicability in spontaneously as well as in mechanically ventilated patients. We identified the best combinations of processing tools applied to minimize estimation errors, and showed the limitations of applying such a technique in presence of experimental noise.

## Additional file

10.1186/s12938-016-0213-y Summarized algorithm to estimate the distribution of ventilation to volume ratios and additional figures showing the standard deviation of simulation results.

## Abbreviations

MBN_2_W: multiple-breath nitrogen washout
FRC: functional residual capacity
EELV: end-expiratory lung volume
$v_{d}$: series dead space
v/V: ventilation-to-volume
MV: mechanical ventilation
$$V_{T}$$: tidal volume
SSE: sum of squared errors
N: number of modeled alveolar units
*ν*: alveolar unit end-expiratory volume
$\gamma$: alveolar unit fraction of tidal volume
J: index of alveolar unit
$$S$$: specific ventilation
$k$: index of breath cycle
$F_{{N_{2} }}^{A}$: alveolar unit concentration of N_2_
$F_{{N_{2} }}^{et}$: end-tidal N_2_ concentration
$F_{{N_{2} }}^{I,A}$: alveolar unit inspired N_2_ concentration
$F_{{N_{2} }}^{I}$: ventilator delivered N_2_ concentration
$V_{T0}$: reference tidal volume
$V_{T}^{I}$: inspiratory tidal volume
$V_{T}^{E}$: expiratory tidal volume
$\beta$: ratio of change in end-expiratory lung volume
$v_{0}$: reference alveolar unit’s end-expiratory volume
λ: gain of smoothness condition
logSD: log-standard deviation
WM: weighting matrix
