# Supplementary material for: Generalized estimation of the ventilatory distribution from the multiple-breath nitrogen washout
Source: Biomed Eng Online. 2016 Aug 2;15:89. doi: 10.1186/s12938-016-0213-y (PMC4970303; doi:10.1186/s12938-016-0213-y)
Supplement: Supplementary file 1 — 10.1186/s12938-016-0213-y Summarized algorithm to estimate the distribution of ventilation to volume ratios and additional figures showing the standard deviation of simulation results. [file 12938_2016_213_MOESM1_ESM.pdf]

Supplement to Generalized estimation of the ventilatory distribution from the multiple-breath nitrogen washout

Gabriel Casulari Motta Ribeiro, Frederico Caetano Jandre, Hermann Wrigge, Antonio Giannella-Neto

Methods

The algorithm to estimate the distribution of ventilation is summarized as follows:

Input

- N alveolar units log distributed on $S(J)$;
- $F_{N_2}^{et}$, V_T^I, V_T^E, v_d and $F_{N_2}^I$ at each breath cycle;
- Reference volume V_{T0} and the EELV at the onset of washout.

Steps

1. Initialize alveolar units and v_d N_2 concentration with the $F_{N_2}^{et}$ measured at baseline.
2. For each breath cycle, calculate each alveolar unit's N_2 concentration using Equation 8.
3. Generate a matrix with rows being breath cycles and columns alveolar units' concentration corrected to the EELV change, like in Equation 10.
4. The ventilation fractions vector (γ) is estimated solving the least square problem in Equation 11, with (or not) the desired constraints (nonnegativity, unitary sum of ventilation fractions and sum of alveolar units' volumes equal to EELV-v_d).

Output

- $\gamma(J)$ for each alveolar unit with respect to a reference tidal volume V_{T0} and the EELV at the onset of washout.

Results

Figures S1 to S4 show the color-coded standard deviation (SD) values of the sum of squared errors (SSE) and the SD from the difference between estimated and reference first three moments (mean, SD and skewness) as a supplementary information to Figures 5 to 8.

The SD of the SSE values depicted in Figure S1 shows that the use of a weighting matrix (WM) has a stabilizing effect on the solutions. The presence of noise generates fast compartments near the limit of the specific ventilation (S) distribution, which are penalized by the WM. As in Figure 5, the smallest SD were obtained with WM and fixed Tikhonov gain (λ) (upper right panel), with minimum effects of using constraints and changing the number of cycles.

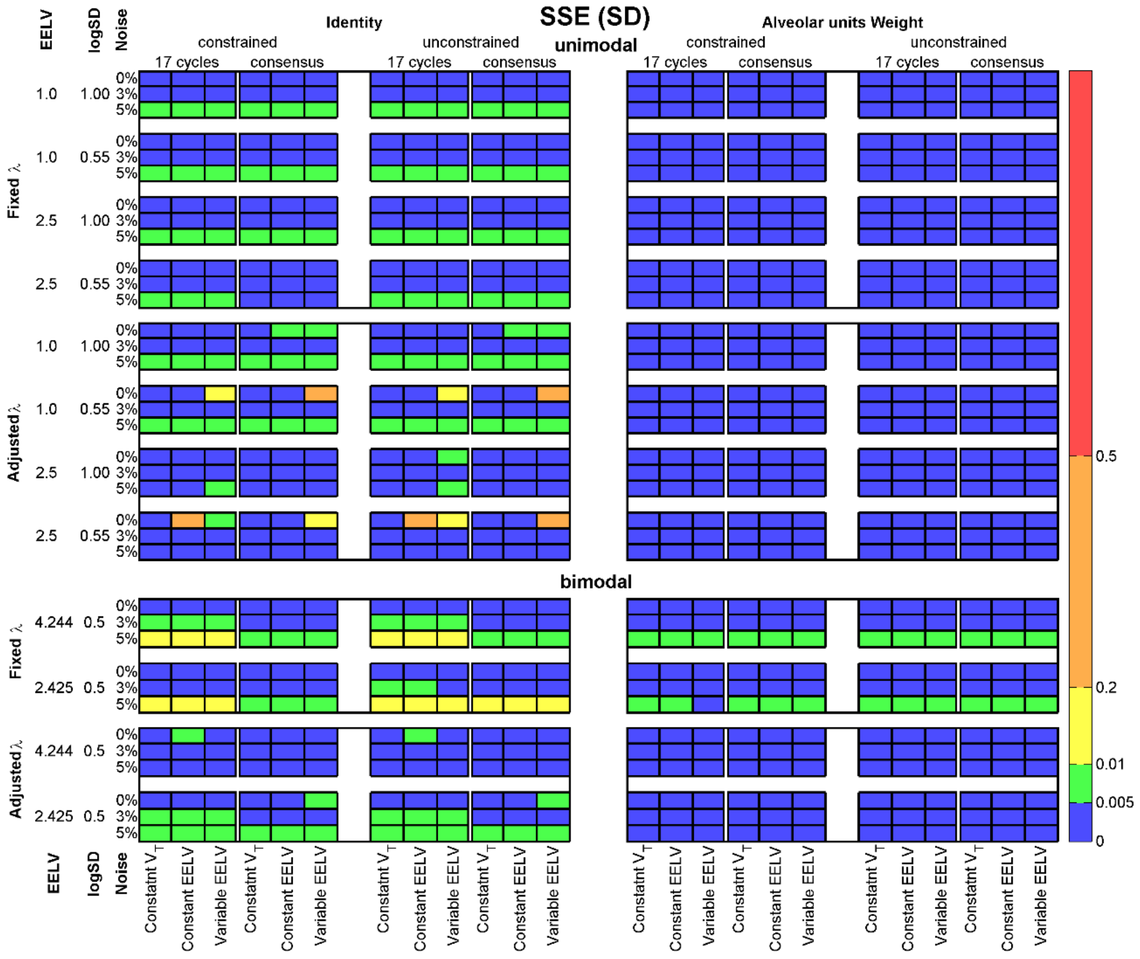

Fig S1 – Standard deviation (SD) of sum of squared errors (SSE) between estimated and reference unimodal and bimodal distributions. All simulation and recovery conditions are depicted and indicated (lines and rows of the matrices). Colors coded on the right side of the figure indicate intervals of values of the SD of SSE; EELV=End-expiratory Lung Volume; V_T = Tidal Volume

Figure S2 presents the SD of the differences between absolute means of estimated and reference unimodal and bimodal distributions. Note the similarity with Figure 6. The most varying solutions are coincident with higher mean differences. Again the best solution was reached with fixed λ and WM. For bimodal distributions, the use of adjusted λ presents a small advantage.

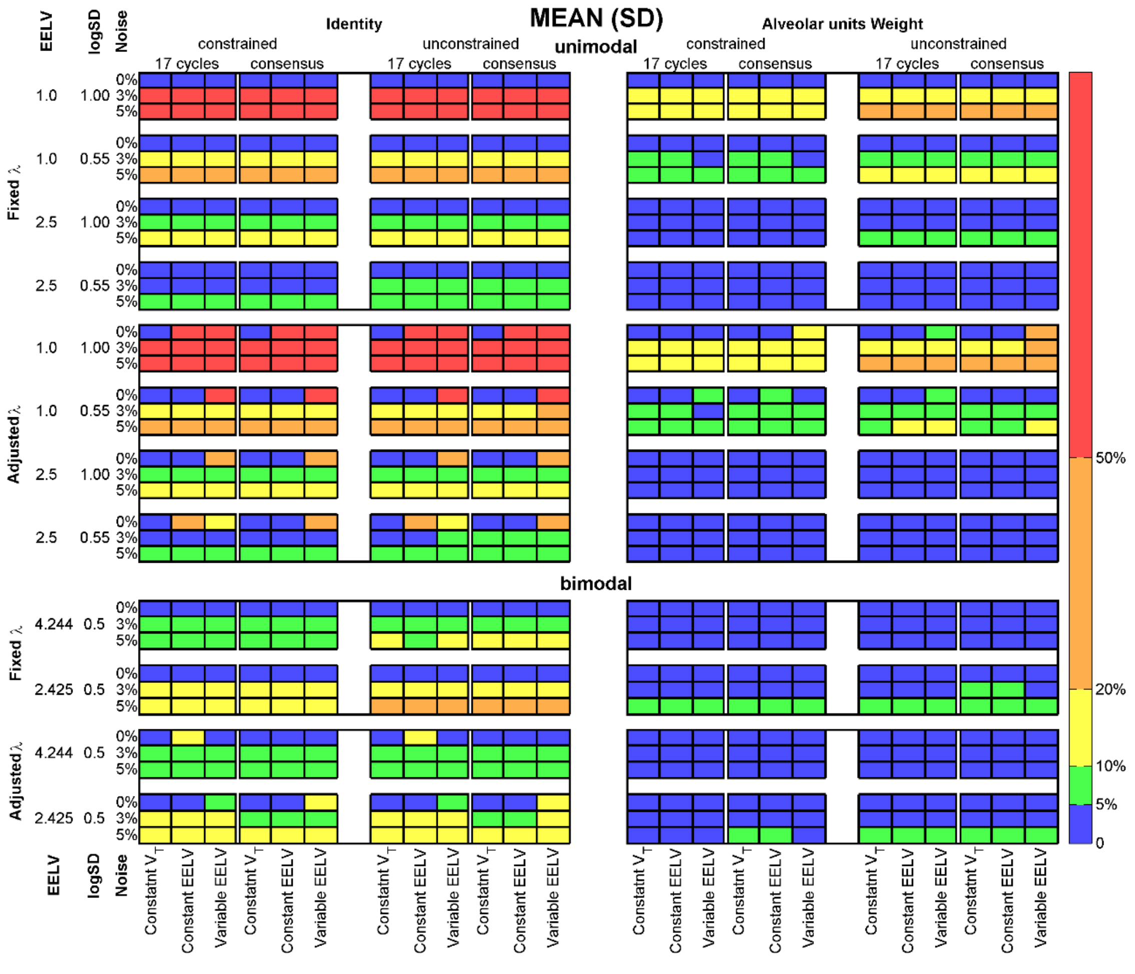

Fig S2 – Standard deviation (SD) of absolute values of the relative differences between means of estimated and reference unimodal and bimodal distributions. All simulations and recovery conditions are depicted and indicated (lines and rows of the matrices). Colors coded on the right side indicate intervals of the SD value. EELV=End-expiratory Lung Volume; V_T = Tidal Volume

As for the mean values of the differences between estimated and reference logSD (Figure 7), their SD are higher when Tikhonov is applied using an identity matrix (see Figure S3). Note that in presence of noise, the use of an adjusted λ leads to less varying solution for both uni- and bimodal distributions. In these cases λ becomes higher, imposing a smoother solution.

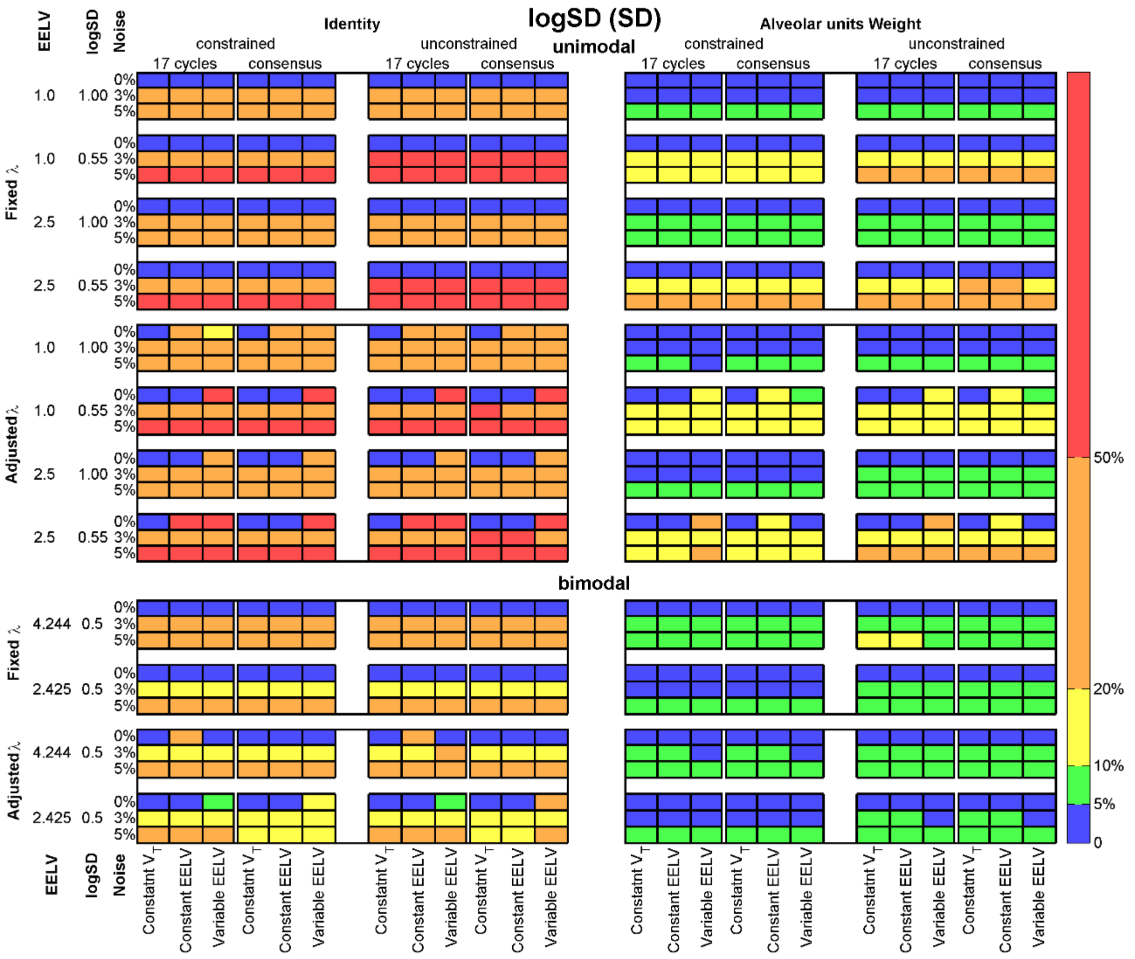

Fig S3 – Standard deviation (SD) of absolute values of the relative differences between logSD of estimated and reference unimodal and bimodal distributions. All simulation and recovery conditions are depicted and indicated (lines and rows of the matrices). Colors coded on the right side of the figure indicate intervals of the SD values. EELV=End-expiratory Lung Volume; V_T = Tidal Volume.

For unimodal distributions, similar to the first two moments, the SD of the difference between estimated and reference skewness are higher for the solutions without the WM (Figure S4). Once more, the smoothed solutions with adjusted λ leads to a less varying skewness estimation. However, the use of a fixed λ gives better error-free solutions and appears to be the best choice.

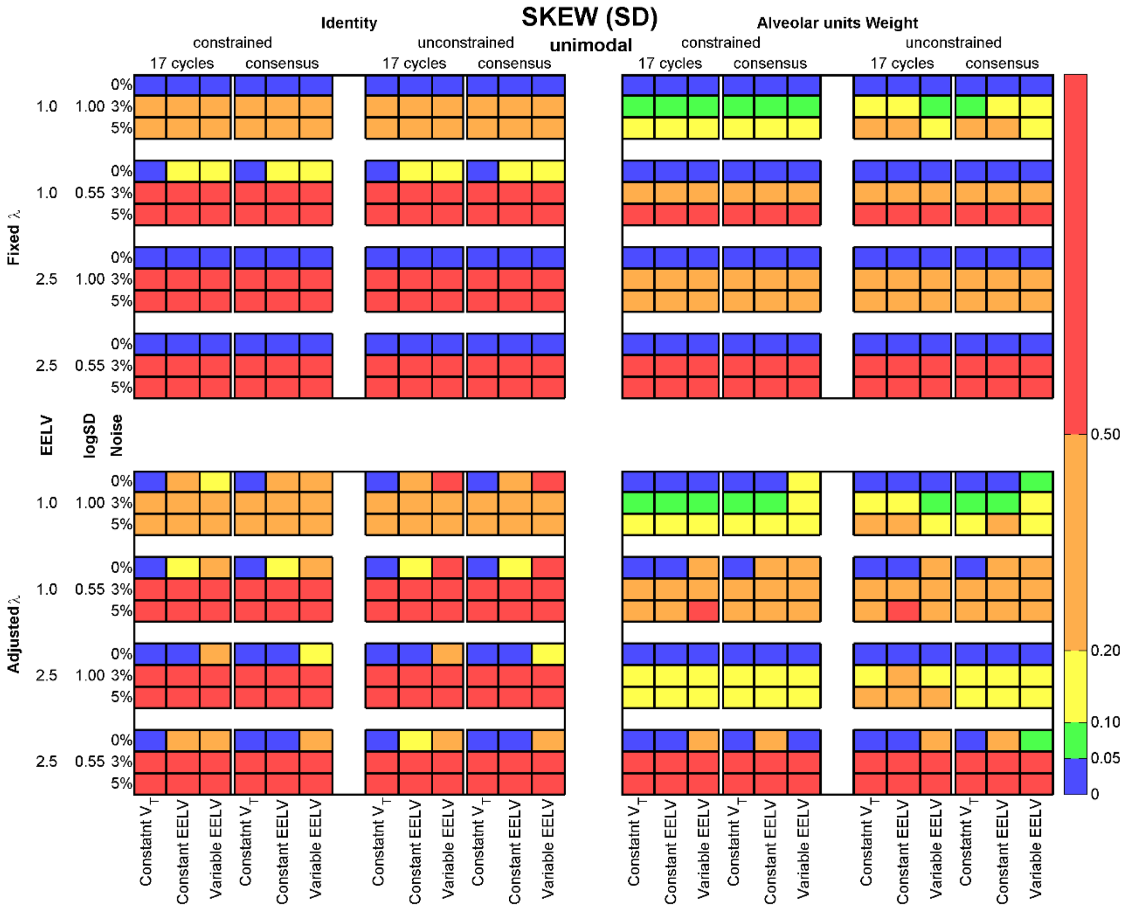

Fig S4 – Standard deviation (SD) of the differences between skewness of estimated and reference unimodal distributions. All simulation and recovery conditions are depicted and indicated (lines and rows of the matrices). Colors coded on the right side indicate intervals of the SD values. EELV=End-expiratory Lung Volume; V_T = Tidal Volume.
